# Research Progress of Chloroquine and Hydroxychloroquine on the COVID-19 and Their Potential Risks in Clinic Use

**DOI:** 10.3389/fphar.2020.01167

**Published:** 2020-07-31

**Authors:** Yan Chen, TaiPeng Shen, LiJun Zhong, ZhiXi Liu, XinWei Dong, TingWenLi Huang, QiuJu Wang, HongTao Xiao

**Affiliations:** ^1^Department of Pharmacy, Sichuan Cancer Hospital & Institute, Sichuan Cancer Center, School of Medicine, University of Electronic Science and Technology of China, Chengdu, China; ^2^Department of Information, Sichuan Cancer Hospital & Institute, Sichuan Cancer Center, School of Medicine, University of Electronic Science and Technology of China, Chengdu, China; ^3^Department of Clinical Laboratory, Sichuan Cancer Hospital & Institute, Sichuan Cancer Center, School of Medicine, University of Electronic Science and Technology of China, Chengdu, China; ^4^Department of Pharmacy, Personalized Drug Therapy Key Laboratory of Sichuan Province, Chengdu, China

**Keywords:** coronavirus, COVID-19, chloroquine, hydroxychloroquine, potential risk

## Abstract

In December 2019, a severe outbreak of a novel coronavirus (COVID-19) occurred in the whole world, posing a great threat to people’s health. With the outbreak and development of the epidemic, how to improve the cure rate, find effective drugs against this virus, has been the most urgent problem. Chloroquine (CQ) was verified effective against COVID-19 *in vitro*. As CQ’s analogue, hydroxychloroquine (HCQ) was also reminded as a potential candidate for treating COVID-19. This review summarizes the latest clinical trials of CQ and HCQ against COVID-19 and its therapeutic regimen in China aiming to share their current usage to the whole world and provide insight into its appropriate future use in the treatment of COVID-19. Through searching the CNKI and Wangfang databases in Chinese language and PubMed, EMBASE, and Ovid databases in English language to identify published reports with the keywords including “coronavirus/COVID, chloroquine, hyroxychloroquine” in alone or combined, we found out the potential preclinical or clinical evidence for using CQ and HCQ against COVID-19. Consequently, we also searched the website of Chinese Clinical Trial Registry (http://www.chictr.org.cn/) till the day on 27^th^, June, 2020. This review found that there are 23 programs aimed to treat the different phases under COVID-19 pipeline in clinic with CQ and HCQ, totally. The inclusion criteria, exclusion criteria and therapeutic regimen were all shared to consult. Among them, seven have been canceled due to lack of patients or other objective factors. There are two trials have completed, which the potential relationship between usage and adverse reactions was discussed emphatically. Through literature research, we suggested that paid close attention to retinal toxicity and ophthalmologic adverse symptom of CQ and HCQ. And the outcome of HCQ in clinic shows better than CQ especially in protective effect with low dosage.

## Introduction

At the end of 2019, a novel coronavirus (CoV) unexpectedly has been detected in Wuhan, a city of China. Then the new coronavirus rapidly infected in every province of China with the passengers traveling (Du et al., 2020). On 12^th^ January, 2020, the world health organization (WHO) named the coronavirus disease as “2019-nCoV”. A few days later, the disease was listed as a public health emergency of international concern. On 11^th^ February, 2020, WHO re-named it as COVID-19 for the disease (Bhagavathula et al., 2020). The new name is taken from the word “corona”, “virus”, and “disease”, with 2019 representing the year that it emerged. On the late June 2020, tens of thousands cases and several thousand deaths have been reported in China alone, while thousands of cases in other countries (Koschmieder et al., 2020). This emerging viral infection was associated with severe human respiratory disease with a ~2–3% fatality rate according to the current report (Erikstrup et al., 2020). Until now, COVID-19 has been reported in South Korea, Japan, Thailand, Malaysia, Singapore, Italy, Unite State America and so on (Lu et al., 2020). Since the outbreak of the COVID-19 in the whole world, the prevention and treatment of the epidemic is severe, complicated, and arduous.

The COVID-19 has been considered to be a new human-infecting betacoronavirus, which shows a high similarity with the genome sequence of severe acute respiratory syndrome (SARS) coronavirus, SARS-CoV and SARS-like CoVs (Coutard et al., 2020). According to reports of the pneumonia cases in China, COVID-19 infection causes severe acute respiratory syndrome with major symptoms such as fever, cough, myalgia, or fatigue. Meanwhile the chest radiographs of patients have been revealed invasive lesions in both lungs (Xie et al., 2020). A great deal of effort has been made to prevent the virus spread rapidly in China. Firstly, most of the provinces in China were launched an emergency response plan for public health emergencies to carry out specific prevention and control measures. Then, 13 cities, such as Wuhan, HuangGang, XiaoGan, and so on, in Hubei province where the virus is most severe, have been made out the strategy of lock-down according to the reports of Hubei provincial health and fitness commission.

In the face of the rapid development of this epidemic, how to improve the cure rate, find effective drugs against the virus, has been the most urgent problem. CQ was found to block COVID-19 infected Vero E6 cells *in vitro* (Al-Bari, 2020), with a 90%-maximal effective concentration (EC_90_) of 6.9 μM (Wang et al., 2020), which can be clinically achievable in rheumatoid arthritis patients who usually received 500 mg administration. Consequently, CQ as an achievable and potential candidate drug to conquer the new coronavirus has been put into action in clinical trials in China. So far, the number of clinical trials with CQ against COVID-19 is fourteen in China, which have never been published before. Naturally, the analog of CQ, hydroxychloroquine (HCQ), shall be gained attention for scientists. Although the price of HCQ is more expensive than CQ, the toxic are lower than CQ (Mackenzie, 1983). Eleven programs of HCQ against COVID-19 have also been carried out in China.

The aim of this paper was to discuss and summarize the progress of effects from CQ and HCQ against COVID-19 and its potential risk in clinic use. We searched the CNKI and Wangfang databases in Chinese language to identify published reports with the keywords used for literature research included “coronavirus, chloroquine, hyroxychloroquine” in alone or in combined. The same way was carried out in PubMed, EMBASE, and Ovid databases in English language. We also searched the website of Chinese Clinical Trial Registry (http://www.chictr.org.cn/ ) till the day on 27^th^, June, 2020. Finally, we hope this study could provide important evidence for rational use of CQ and HCQ against this unexpected disease.

## Results and Discussion

### Brief Introduction of the Origin of CQ and HCQ

**Figure 1:**
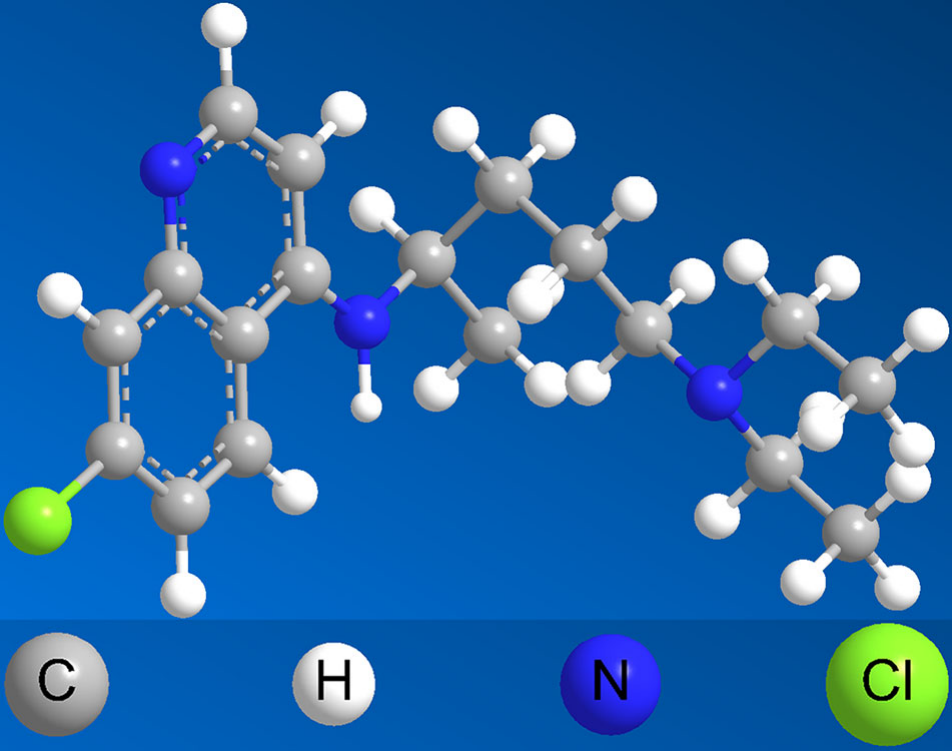
The 3D structure of CQ.

**Figure 2:**
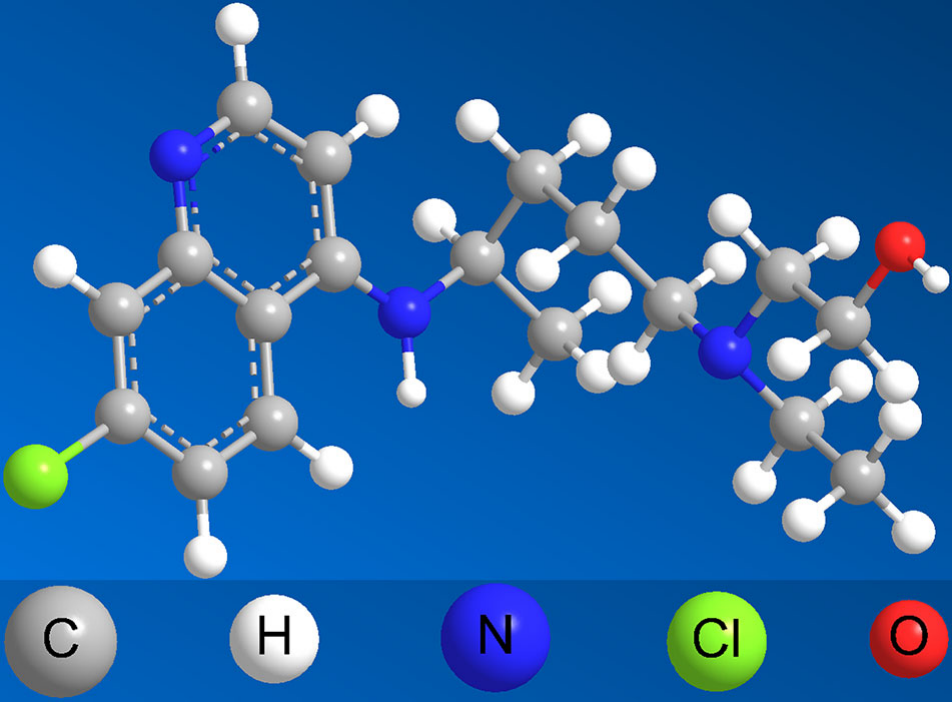
The 3D structure of HCQ.

CQ was originated from quinine, which was short-acting alkaloid drug extracted from cinchona bark and was the first drug used widely for malaria from the 19th century (Ross, 1918). Quinine remained the mainstay of treating malaria until the 1920s with the obstacle of its poor tolerability, poor compliance with complex dosing regimens and adverse effects with its use (Organization, 1990). Due to these challenges, CQ, as the most important derivate from quinine, was extensively used, especially beginning in the 1940s (Yakoub AdenAbdi OE et al., 1995). With extensive use, the adverse reaction of CQ, especially in retinal toxicity and ophthalmologic adverse symptom was obvious (Marmor et al., 2016). Then HCQ, which aimed to is proved to be threefold less toxic than CQ (Mackenzie, 1983) was designed. Both of the two drugs have a flat aromatic core structure and are weak bases due to the presence of a basic side chain. The basic side chain is thought to contribute to the accumulation of these drugs in intracellular compartments, especially lysosomal compartments, which verifies to be crucial for the potential interaction with nucleic acids (Achan et al., 2011). The 3D structure of CQ and HCQ is exhibited in **Figures 1** and **2**. Neither CQ nor HCQ underwent conventional drug developments, the usage of them were broaden to anti-virus, anti-cancer, anti-rheumatoid arthritis, and so on.

### Brief Introduction of CQ on the Virus

**Figure 3:**
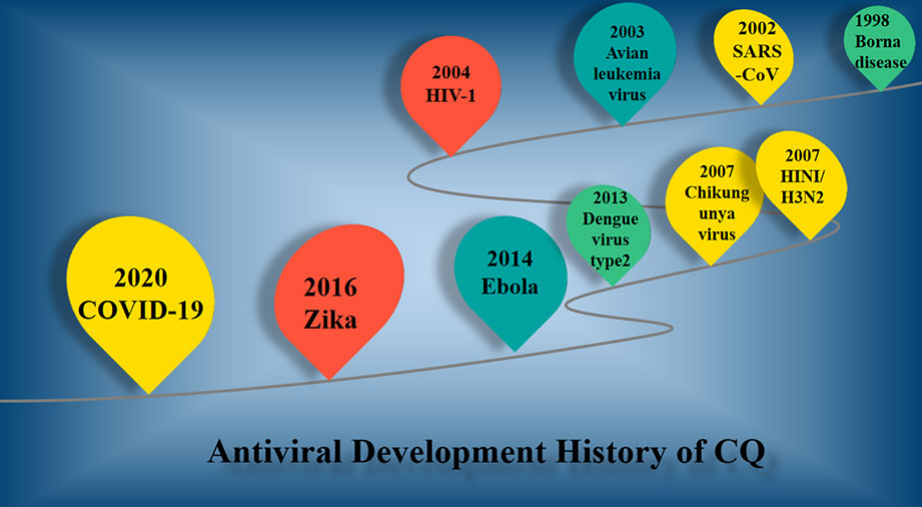
The antiviral development history of CQ.

CQ is considered to be an antimalarial drug for so many years, which clinic history could be traced back to 1820 (Chatre et al., 2018). The 3D structure of CQ is shown in **Figure 1**. With French famous pharmacists successfully extracting from cinchona, quinine is the earliest antimalarial for treatment of febrile diseases in the history. In 1930’s, German scientists synthesized the CQ with lower toxicity and stronger effect than quinine (White et al., 1983). The development of CQ against virus was shown in **Figure 3**, meanwhile the potential mechanism was exhibited correspondingly in **Table 1**. As shown in **Table 1** and **Figure 3**, the group of Daniel Gonzalez-Dunia used CQ in 1998 (Gonzalez-Dunia et al., 1998), as a lysosomotropic agent, to inhibit the borna disease virus infection. The mechanism of borna disease virus entry into C6 rat glioma cells were involved in pH-dependent fusion after internalization of the virion by endocytosis, while CQ could prevent endosomal acidification to inhibit the entry of borna disease virus. At the end of 2002, SARS coronavirus was broken out (Casey et al., 2006). Consequently, CQ was verified effect effectively to inhibit the infection and transmission of SARS-CoV *via* increasing endosomal pH, as well as interfering with the glycosylation of cellular receptors of SARS-CoV (Vincent et al., 2005). Also, the similar function was observed in avian leukemia virus (ALV) by CQ (Diaz-Griffero et al., 2002), which accumulates in endocytic compartments and increases the endosomal pH to reduce the entry of pseudotyped ALV type B. Then in 2006, CQ had been shown to be effective in human immunodeficiency virus type 1 (HIV) (Savarino et al., 2006). With the progress of experimental technology and methodological approaches, the effect of CQ *via* autophagy inhibition were got extensive attention worldwide (Shi et al., 2017). The resurgence of Chikungunya virus (CHIKV) in India and the Indian Ocean islands associated with severe clinical symptoms also made great public health concern in 2007 (Lo Presti et al., 2014). After a series of experiments, the scientists in India suggested the mechanism of CQ to inhibit the CHIKV infection in Vero cells involving the early stages of infection in 2010 (Khan et al., 2010). And the scientists confirmed that CQ could block the production of proinflammatory cytokines and the proliferation of monocytes, macrophages, and lymphocytes (Paton et al., 2011). The next year, a randomized, double-blind, placebo-controlled clinic trial from Singapore (registration number: NCT01078779) (Di Trani et al., 2007) have verified that CQ did not show protective efficacy against influenza infection (HIN1), although CQ was confirmed viable against both H1N1 and H3N2 influenza strains *in vitro* (Farias et al., 2013). Soon afterwards, CQ was test to affect intracellular exocytic pathways by increasing endosomal pH against dengue virus type 2 (DENV-2). The group of Farias KJ verified that 50 μg/ml of CQ could significantly inhibit of DENV-2 load in infected Vero and C6/36 cells production in 2013 (Farias et al., 2013). The large outbreak of Ebola virus (EBOV) disease in parts of West Africa which was first recognized in March 2014 (Gire et al., 2014). CQ was honorably repurposed as a potential candidate for treating EBOV. Although it was suggest the replication of EBOV was impaired by CQ *in vitro*, it might not be affected *in vivo*, especially in EBOV-infected guinea pig model (Dowall et al., 2015). CQ can reduce the number of Zika-infected cells *in vitro*, and inhibits virus production and cell death promoted by Zika infection without cytotoxic effects (Delvecchio et al., 2016). Then it has been shown in the mice model that CQ can cut off Zika virus from the mother-fetal pathway of vertical infection in 2016 (Delvecchio et al., 2016). Up to now, CQ was verified effective against COVID-19 *in vitro* (Carrière et al., 2020) *via* influencing bis (monoacylglycero) phosphate entry through hijacking of the endocytic pathway.

**Table 1:** The development of CQ against different type of virus and main mechanism behind the effect.

Drug	Virus	Cells	Main mechanism	Year	Ref
CQ	Borna disease virus	C6 rat glioma	The mechanism of borna disease virus entry into C6 rat glioma cells were involved in pH-dependent fusion after internalization of the virion by endocytosis, while CQ could prevent endosomal acidification to inhibit the entry of borna disease virus.	1998	(Gonzalez-Dunia et al., 1998)
CQ	SARS-CoV	Vero,Vero E6	CQ inhibited the infection and transmission of SARS-CoV *via* increasing endosomal pH, as well as interfering with the glycosylation of cellular receptors of SARS-CoV.	2002–2006	(Keyaerts et al., 2004; Savarino et al., 2004; Achan et al., 2011)
CQ	Avian leukemia virus	293T, DF-1 chicken embryonic fibroblasts	CQ accumulated in endocytic compartments and increases the endosomal pH to reduce the entry of pseudotyped ALV type B.	2003	(Diaz-Griffero et al., 2002)
CQ	HIV-1	MT-4	CQ has been shown to interfere with viral replication by impairing the glycosylation machinery in the Golgi that would direct trafficking and maturation of nascent viral proteins.	2004–2006	(Savarino et al., 2004; Savarino et al., 2006)
CQ	Chikungunya virus	Vero	The mechanism of CQ to inhibit the CHIKV infection in Vero cells involving the early stages of infection	2007-2010	(Khan et al., 2010)
CQ	Influenza infection (HIN1)	A549	Although CQ active against HIN1 *in vitro*, it did not prevent the weight loss associated with HIN1 infection in mice after challenge with viruses expressing an H1 or H3 hemagglutinin protein.	2007	(Di Trani et al., 2007; Vigerust and McCullers, 2007)
CQ	Influenza infection (H3N2)	MDCK	There was a clear correlation between the EC_50_ of CQ *in vitro* and the electrostatic potential of the HA subunit mediating the virus/cell fusion process.	2007	(Di Trani et al., 2007)
CQ	Dengue virus type 2	Vero, C6/36 cells	CQ affected intracellular exocytic pathways by increasing endosomal pH against dengue virus type 2.	2013	(Farias et al., 2013)
CQ	Ebola virus	Vero, Vero 76	CQ and related compounds must affect a process downstream of cell binding. These observations are consistent with CQ arresting endosomal trafficking from the early to late endosome, which causes accumulation of virus that does not progress to the late endosome as normal, resulting in an abortive infection.	2014	(Madrid et al., 2013)
CQ	Zika virus	Vero, human brain microvascular endothelial cells, human neural stem cells, and mouse neurospheres	CQ reduces the number of Zika-infected cells *in vitro*, and inhibits virus production and cell death promoted by Zika infection without cytotoxic effects. In addition, CQ treatment partially reveres morphological changes induced by Zika infection in mouse neurospheres.	2016	(Delvecchio et al., 2016)
CQ	COVID-19	Vero E6	CQ could influence the bis (monoacylglycero) phosphate entry through hijacking of the endocytic pathway to affect viral replication.l	2020	(Carrière et al., 2020)

### The Clinical Trials of CQ Against COVID-19 in China

Based on the urgent need of current clinical treatment, on 18^th^ February 2020, the general office of the national health commission of China published the “new coronavirus pneumonia diagnosis and treatment guideline (the sixth edition)” to increase CQ into the section of antiviral treatment drugs within this guideline. Here, we list the clinical trials of CQ against COVID-19 in China. There are 14 programs aimed to treat the various degree of COVID-19 in clinic, which divided by clinic classification. The clinic classifications are list as: the mild, normal, severe, and critical type. Consequently, the specific define of the above types of patients with COVID-19 are displayed:

(1) The mild type of patients with COVID-19.

The clinical symptoms were mild, and there was no sign of pneumonia on lung imaging.

(2) The normal type of patients with COVID-19.

Patients with fever, or/and respiratory symptoms, meanwhile the lung imaging with pneumonia.

(3) The severe type of patients with COVID-19.

In accordance with any of the following clinic symptoms: Shortness of breath, RR≥30 times/min; In resting state, oxygen saturation is less than 93%; Partial arterial oxygen pressure (Pa0_2_)/absorption oxygen concentration (Fi0_2_)≤300mgHg (The areas with high altitudes (over 1000 meter) shall be corrected for Pa0_2_/Fi0_2_ according to the following formula: Pa0_2_/F10_2_ x[atmospheric pressure (mmHg)/760]); The lesion progression with pulmonary imaging more than 50% within 24 to 48 h.

(4) The critical type of patients with COVID-19.

Meet one of the following clinic symptoms: Respiratory failure, mechanical ventilation; Shock; Combined with other organ failure, requiring intensive care unit.

Until now, there are 14 clinic trials of CQ against COVID-19 in China supplying in **Table 2**, which aimed to cover the all clinic classification of COVID-19. The details of inclusion criteria and exclusion criteria were supplied in the **Supplementary Table 1**, which could provide convenient reference for CQ-related clinical trials against COVID-19 in the near future. Among those above mentioned clinic trails, four were canceled by the investigator due to some objective factors. Herein, 10 programs are still ongoing. The duration of study therapy varies from one study to another (from 3 to 12 months). All the type of the clinical trial belongs to the “Intervention study”. Obviously, we focus on the specific dosage of CQ in the treatment of COVID-19 either in alone or in combination usage. Only three (3/14, 21.43%) studies have provided the usage and dosage of CQ against COVID-19. The one route is *via* inhaled route (registration number: ChiCTR2000029975), the others are by oral administration (registration number: ChiCTR2000029992 and ChiCTR2000030054). The regimen of CQ in the inhaled administration is 150 mg/q12h, which could be inferred that 300 mg per day of CQ could be into the blood without the loss caused by gastro-intestinal tract. However, the regimen of CQ *via* the oral administration was 1g for 2 days, and from the 3^rd^ day was 0.5 g for 12 days. Basing on these details reported in the studies, the dosage of CQ against COVID-19 is at least above 300 mg per day and 500 mg per day *via* inhaled administration and oral administration, respectively, within treating cycle from 7 to 14 days. Although the outcomes of these clinic trials are not declared because of these programs are still ongoing, most of these studies have promised that the time of sharing individual participant data (IPD) will be within 6 months after the trial complete.

**Table 2:** The list of clinical trials of CQ against COVID-19 in China.

Number	Registration number	Registration name	Type of clinical trial	Registration date	Group		Progress	Anticipated execute time
					Intervention	Comparator		
1	ChiCTR2000029609	A prospective, open, multicenter clinical study on the treatment of COVID-19 with chloroquine phosphate	Intervention study	6/2/2020	Mild-moderate chloroquine group: chloroquine phosphate was taken by oral Mild-moderate combination group: chloroquine phosphate plus Lopinavir/ritonavir was taken by oral Severe-chloroquine group: chloroquine phosphate was taken by oral	Mild-moderate group Lopinavir/ritonavir group: oral Lopinavir/ritonavir Severe-group Lopinavir/ritonavir group: oral Lopinavir/ritonavir	Not yet recruiting.	From 2020-02-10 To 2020-12-31. The time of sharing IPD will be within 6 months after the trial complete.
2	ChiCTR2000029826	A randomized, double-blind, parallel-controlled study of chloroquine phosphate combined with standard therapy versus standard therapy for severe/critical COVID-19	Intervention study	14/2/2020	Phosphoric chloroquine: two tablets phosphoric chloroquine bid	Placebo two tablets placebo bid	Canceled by the investigator	From 2020-02-17 To 2020-03-17
3	ChiCTR2000029837	A randomized, double-blind, parallel-controlled study of chloroquine phosphate combined with standard therapy versus standard therapy for mild/normal COVID-19	Intervention study	15/2/2020	Phosphoric chloroquine: two tablets phosphoric chloroquine bid	Placebo two tablets placebo bid	Canceled by the investigator	From 2020-02-17 To 2020-03-17
4	ChiCTR2000029935	Single-arm clinical study on the treatment of COVID-19 with chloroquine phosphate	Intervention study	16/2/2020	Treated with conventional treatment combined with chloroquine phosphate	Conventional therapy	Recruiting	From 2020-02-06 To 2021-02-06 The time of sharing IPD will be within 6 months after the trial complete.
5	ChiCTR2000029939	A single blind, randomized, controlled clinical trial of chloroquine phosphate tablets in the treatment of COVID-19	Intervention study	16/2/2020	Conventional treatment with chloroquine phosphate	The conventional treatment group will be treated according to the guidance of the “Diagnosis and Treatment Scheme of COVID-19” published by the National Health Commission	Recruiting	From 2020-02-06 To 2021-02-06 The time of sharing IPD will be within 6 months after the trial complete.
6	ChiCTR2000029975	Single-arm clinical study on the treatment of COVID-19 with chloroquine phosphate	Intervention study	18/2/2020	In addition to the routine treatment, add 150 mg chloroquine phosphate dissolved in 5 ml of normal saline, q12h, inhaled by atomization for 1 week.	Conventional therapy	Not yet recruiting.	From 2020-02-24 To 2020-05-31 The time of sharing IPD Within 6 months after the trial complete
7	ChiCTR2000029988	Chloroquine phosphate treatment for severe COVID-19	Intervention study	18/2/2020	Chloroquine Phosphate	Conventional therapy	Recruiting	From 2020-02-13 To 2020-05-31 The time of sharing IPD will be within 6 months after the trial complete.
8	ChiCTR2000029992	A prospective, open randomized controlled trial of chloroquine phosphate and hydroxychloroquine sulfate in patients with COVID-19	Intervention study	18/2/2020	Hydroxychloroquine sulfate gloup: Hydroxychloroquine sulfate 0.2 g bid for 14 days Chloroquine phosphate gloup: The first dose of chloroquine phosphate was 1 g for 2 days, and the 3^rd^ day was 0.5 g for 12 days	Recommended treatment plan for novel coronavirus pneumonia severe and critical cases	Not yet recruiting.	From 2020-02-17 To 2020-05-20 The time of sharing IPD will be within 6 months after the trial complete.
9	ChiCTR2000030031	A randomized, double-blind, parallel-controlled study of chloroquine phosphate combined with standard therapy versus standard therapy for mild/normal COVID-19	Intervention study	21/2/2020	Phosphoric chloroquine: two tablets phosphoric chloroquine bid	2 tablets placebo BID	Canceled by the investigator	From 2020-02-20 To 2021-03-20
10	ChiCTR2000030054 (Retrospective registration)	An open randomized controlled trial for Chloroquine phosphate and Hydroxychloroquine sulfate in the treatment of mild and common novel COVID-19	Intervention study	22/2/2020	Hydroxychloroquine sulfate gloup: Hydroxychloroquine sulfate 0.2g bid for 14 days Chloroquine phosphate gloup: The first dose of chloroquine phosphate was 1 g for 2 days, and the 3^rd^ day was 0.5 g for 12 days	Recommended treatment plan for novel coronavirus pneumonia diagnosis and treatment plan	Not yet recruiting.	From 2020-02-22 To 2020-05-21 The time of sharing IPD will be within 6 months after the trial complete.
11	ChiCTR2000030417	Efficacy and safety of chloroquine phosphate inhalation combined with standard therapy in the treatment of novel COVID-19	Intervention study	01/3/2020	Combined standard therapy of chloroquine phosphate aerosol inhalation solution	Water for injection atomization inhalation combined with standard therapy	Canceled by the investigator	From 2020-03-01 To 2020-06-30
12	ChiCTR2000030718 (Retrospective registration)	Randomized controlled trial for Chloroquine Phosphate in the Treatment of novel COVID-19	Intervention study	11/3/2020	Chloroquine phosphate	Conventional therapy	Recruiting	From 2020-02-12 To 2020-05-30 The time of sharing IPD Real time access
13	ChiCTR2000030987	Clinical Trial of Favipiravir Tablets Combine With Chloroquine Phosphate in the Treatment of novel COVID-19	Intervention study	20/3/2020	Experimental group 1: the oral trial drug favipiravir tablets plus chloroquine phosphatetablets tablets group Experimental group 2: the oral trial drug favipiravir tablet	Placebo group	Recruiting	From 2020-03-05 To 2020-06-25 The time of sharing IPD will be within 6 months after the trial complete.
14	ChiCTR2000031204 (Retrospective registration)	A multicenter, single-blind, randomized controlled clinical trial for chloroquine phosphate in the treatment of novel COVID-19	Intervention study	24/3/2020	Chloroquine phosphate tablets *via* oral	Placebo	Recruiting	From 2020-01-30 To 2020-04-30 The time of sharing IPD will be within 6 months after the trial complete.

bid, bis in die; IPD, information of participant data.

### Potential Risk of CQ in Clinic to Treat COVID-19

According to the “Expert consensus on chloroquine phosphate for the treatment of novel coronavirus pneumonia” edit by The multicenter collaboration group of department of science and technology of guangdong province and health commission of guangdong province for chloroquine in the treatment of novel coronavirus pneumonia (pneumonia., 2020), the dosage and treatment plan of CQ against COVID-19 are 500 mg/bid within a treating cycle of 10 days. If severe gastrointestinal reactions appear, the dosage should be reduced to 500 mg/qd or even stopping administration. If the nucleic acid from the pharynx swab turns negative and lasts for at least 3 days as negative during the CQ treatment, the plan of withdrawal can be considered. But the minimum program of CQ treatment requires at least 5 days.

Based on previous reports, CQ is rapidly absorbed from the gastro-intestinal tract when administration *via* oral route (Essien et al., 1988). The average oral bioavailability is approximately 89% with a relatively uncertain half-life as long as 5-60 days (Ducharme and Farinotti, 1996), with a median half-life of 21-30 days. The clinical lethal dose of CQ was reported as 3-5 g according to the reports by New England Journal Medicine in 1988 (Riou et al., 1988). If the adverse reactions from CQ emerged, the combining early mechanical ventilation with diazepam and epinephrine *via* intravenous infusion might be effective way to treat CQ poisoning (Samanidou et al., 2005). Furthermore, ammonium chloride can be used to accelerate excretion and reduce the concentration of CQ in plasma; owing to CQ is an alkaloid (Samanidou et al., 2005).

What’s more, reviewing the past clinical experience by CQ against antiviral, it might be emerged unexpected results contrary to our wish. For example, as above mentioned, CQ exhibited a good antiviral effect on MDCK cells infected with human influenza a virus H1N1 and H3N2, while the clinical trial (registration number: NCT01078779) have not further confirmed this result. This clinical trial recruited 1516 cases of H1N1 eligible participants (Paton et al., 2011). In this trial, CQ was administered at the dosage of 500 mg/qd for the 1^st^ week. Then the participants were taken 500 mg once a week to complete a total course of 12 weeks. The data confirmed that CQ did not prevent infection with influenza, while the incidence of adverse reactions was higher (P < 0.0001). The adverse reactions containing headache, dizziness, nausea, diarrhea, and blurred vision were verified positive with CQ. Herein, the treating regimen of CQ against COVID-19 in China might be at risk and called for more monitoring. A blood sample concentration of CQ suggests monitoring continuously.

### Brief Introduction of HCQ on the Virus

The main development of HCQ against virus was very similar with CQ, owing to the similarity from chemical structure. In other words, if CQ played positive effect against some specific virus, HCQ would be considered naturally and studied simultaneously. The 3D structure of HCQ was exhibited in **Figure 3**. Indeed, HCQ was also verified the activity of antivirus, such as HIV-1 (Chiang et al., 1996), SARS-CoV (Biot et al., 2006), Zika Virus (Kumar et al., 2018), CHIKV (Mathew et al., 2017), DENV (L. F. Wang et al., 2015), COVID-19 (Hashem et al., 2020), and so on. Consequently, we shall pay more attention to the usage and dosage of HCQ in clinic, especially against COVID.

### The Clinical Trials of HCQ Against COVID-19 in China

Similarly, we searched the website of the Chinese clinical trials registry and found that there are currently eleven approved clinical studies under way, which contain two repetitive studies within in CQ-related trials (registration number: ChiCTR2000029992 and ChiCTR2000030054) and showing in **Table 3**. The details of inclusion criteria and exclusion criteria of HCQ-related trails were also supplied in the **Supplementary Table 2**, which would be also helpful for clinicians to consult. As shown in **Table 3**, there is only one program (1/11, 9.09%) aiming to “observational study”. Ten programs (10/11, 91.01%) are focused on the “intervention effect of HCQ against COVID-19”. In fact, three studies have canceled by the investigator due to lack of eligible participants. The anticipated execute time of HCQ-related study varies from 1 to 6 months, which are shorter than the trials of CQ-based treatment study. Back to the point on dosage of HCQ against COVID-19, five studies (5/11, 45.45%) supply the accurate dose of HCQ among these clinic trials. In the study of ChiCTR2000029559 and ChiCTR2000029740, 0.2 g bid of HCQ *via* oral administration are given. The maximum dose of HCQ can be calculated as 400 mg per day without the key factor, that is, specific treating cycle. In the study of ChiCTR2000029992 and ChiCTR2000030054, the duration time of HCQ against COVID is exhibited, that is 14 days, with the similar regimen comparing with study of ChiCTR2000029559 and ChiCTR2000029740.

**Table 3:** The list of clinical trials of HCQ against COVID-19 in China.

Number	Registration number	Registration name	Type of clinical trial	Registration date of clinical trial	Group		Progress	Anticipated execute time
					Intervention	Comparator		
1	ChiCTR2000029559	Hydroxychloroquine sulfate in patients with COVID-19	Intervention study	4/2/2020	Hydroxychloroquine 200 mg bid *via* oral	Placebo group: Starch pill bid *via* oral	Complete	From2020-01-31 To 2020-02-29 The time of sharing IPD Within 6 months after the trial complete
2	ChiCTR2000029740	Study on the effectiveness of hydroxychloroquine sulfate in treating COVID-19	Intervention study	11/2/2020	Hydroxychloroquine 200 mg bid *via* oral	Conventional therapy	Recruiting	From 2020-02-11 To 2020-02-29 The time of sharing IPD Within 6 months after the trial complete
3	ChiCTR2000029760	Study on the effectiveness of hydroxychloroquine sulfate in treating mild/normal COVID-19	Intervention study	12/2/2020	Hydroxychloroquin	Lopinavir/Ritonavir	Canceled due to lack of patients.	From 2020-02-12 To 2020-08-11
4	ChiCTR2000029761	Study on the effectiveness and safety of hydroxychloroquine sulfate in treating mild/normal COVID-19	Intervention study	12/2/2020	Low-dose group:Low-dose hydroxychloroquine and conventional therapy Medium-dose group:Medium-dose hydroxychloroquine and conventional therapy High-dose group:High-dose hydroxychloroquine and conventional therapy	Conventional therapy	Canceled due to lack of patients.	From 2020-02-13 To 2020-04-30
5	ChiCTR2000029762	Study on the effectiveness and safety of hydroxychloroquine sulfate in treating severe/critical COVID-19	Intervention study	12/2/2020	Conventional treated with hydroxychloroquine	Conventional treatment	Canceled due to lack of patients.	From 2020-02-12
6	ChiCTR2000029868	Multicenter clinical study on the treatment of COVID-19 with hydroxychloroquine sulfate	Intervention study	15/2/2020	Hydroxychloroquine sulfate 1200 mg daily for 3 days followed by a maintenance dose of 800 mg daily (total treatment duration: two or 3 weeks for patients with mild to moderate or severe disease, respectively).	Conventional treatment	Complete	From 2020-02-06 To 2020-06-30 The time of sharing IPD Within 6 months after the trial complete
7	ChiCTR2000029992	A prospective, open randomized controlled trial of chloroquine phosphate and hydroxychloroquine sulfate in patients with COVID-19	Intervention study	18/2/2020	Chloroquine phosphate gloup: Chloroquine phosphate 1.0 g with 2 days for the first dose, 0.5 g for 12 days from the 3^rd^ day Hydroxychloroquine sulfate gloup: Hydroxychloroquine sulfate 0.2 g BID for 14 days	Recommended treatment plan for severe and critical cases in COVID-19	Not yet recruiting.	From2020-02-17 To 2020-05-20 The time of sharing IPD Within 6 months after the trial complete
8	ChiCTR2000030054	An open randomized controlled trial for Chloroquine phosphate and Hydroxychloroquine sulfate in the treatment of mild and common novel COVID-19	Intervention study	22/2/2020	Hydroxychloroquine sulfate gloup: Hydroxychloroquine sulfate 0.2 g BID for 14 days Chloroquine phosphate gloup: The first dose of chloroquine phosphate was 1 g for 2 days, and the 3^rd^ day was 0.5 g for 12 days	Recommended treatment plan for novel coronavirus pneumonia diagnosis and treatment plan	Not yet recruiting.	From 2020-02-22 To 2020-05-21 The time of sharing IPD Within 6 months after the trial complete
9	ChiCTR2000031174	Effectiveness and safety of hydroxychloroquine sulfate in the preventive treatment of novel COVID-19	Intervention study	23/3/2020	Hydroxychloroquine	Placebo	Not yet recruiting.	From 2020-03-23 To 2020-09-30 The time of sharing IPD Within 6 months after the trial complete
10	ChiCTR2000031782	A questionnaire investigation of hydroxychloroquine for its potential protective effect against novel COVID-19	Observational study	10/4/2020	/	/	Not yet recruiting.	From 2020-04-10 To 2020-06-30 The time of sharing IPD Within 6 months after the trial complete
11	ChiCTR2000032487	Study for using sulfate in the prevention and control of novel COVID-19 in high and low prevalence communities	Intervention study	29/4/2020	Hydroxychloroquine	Placebo	Not yet recruiting.	From 2020-03-01 To 2020-09-30 The time of sharing IPD Within 6 months after the trial complete

bid, bis in die; IPD, information of participant data.

To our relief, there are two trials, which have been published on 10^th^, April, 2020 from Chen and co-workers (registration number: ChiCTR2000029559) (Chen Z. et al., 2020) and 14^th^, May, 2020 from the group of Tang (registration number: ChiCTR2000029868) (Tang et al., 2020), respectively.

In the trial of ChiCTR2000029559, 62 patients who met the trial criteria were randomly assigned in two groups. Patients in both groups received the standard treatment (oxygen therapy, antiviral agents, antibacterial agents, and immunoglobulin, with or without corticosteroids) as the authors depicted. The patients in the HCQ treatment group received additional oral HCQ for 400 mg/d (200 mg/bid) for 5 days continuously, while patients in the control group just with the standard treatment only. The results showed that pneumonia was improved in 67.7% (42/62) of patients, with 29.0% moderately absorbed and 38.7% significantly improved verified by chest CT. For adverse effects, it should be noted that there were two patients with mild adverse reactions in the HCQ treatment group. The one patient developed rash, the other one experienced a headache. None severe side effects appeared in HCQ treating group. This trial did give us an exciting outcome, while the small number of participants and short of duration time was the disadvantage in this study.

In the trial of Tang’s colleagues (Tang et al., 2020), HCQ is administrated at a loading dose of 1200 mg daily for 3 days followed by a maintenance dose of 800 mg daily during 2 or 3 weeks for patients with mild to moderate or severe type of patients with COVID-19, respectively. Totally, 150 patients have been recruited; in which 148 belong to mild to moderate type of patients with COVID-19 and two were geared to severe type of patients with COVID-19. As the result, the treatment of HCQ did not result in a significantly higher probability of negative conversion than standard of care alone in patients admitted to hospital with mainly persistent mild to moderate COVID-19. To make matters worse, adverse events were higher in HCQ recipients than in non-recipients. The most common adverse event in the HCQ recipients was diarrhoea, reported in 7/70 (10%) patients. Other non-serious adverse events as the authors depicted were vomiting 2/70 (2.86%), kidney injury 1/70 (1.43%), nausea 1/70 (1.43%), abdominal discomfort 1/70 (1.43%), sinus bradycardia 1/70 (1.43%), hypertension 1/70 (1.43%), and so on, which never been found in HCQ non-recipients. Two HCQ recipients reported serious adverse event, such as disease progression and upper respiratory tract infection. It’s worth noting that the usage of HCQ was in combination with antiviral agents (arbidol, virazole, lopinavir/ritonavir, oseltamivir, entecavir) rather than in alone in this trial. Herein, the drug interaction needs to be considering further, while this trial without the relevant design about the relationship between HCQ and antiviral agents. Basing on some reports, the lopinavir/ritonavir, atazanavir/ritonavir may increase exposure by inhibition of CYPs enzymes 2C8, 3A4, and 2D6. Consequently, increase the risk of QTc interval prolongation when concurrent use with HCQ (Abena et al., 2020).

According to the result of literature research, we found an article written in Chinese language, which publish a latest clinic trial about HCQ (registration number: NCT04261517) (Chen J. et al., 2020). Herein, we shall also share the usage and dosage about this study. Only 30 patients confirmed with mild type or normal type of COVID-19 from Shanghai, China, were enrolled in this study. Patients were randomly assigned in two groups, that is, with or without HCQ treatment group. The treating regimen was that 400 mg qd of HCQ for 5 days. The total dosage of HCQ was similar with Chen’s study (Chen Z. et al., 2020), while the frequency was different. The findings from this study indicated that there were no significant differences in the improvement of negative conversion between standard of care alone group and HCQ treatment group. What’s more, the incidence of gastrointestinal adverse reactions is higher in HCQ treatment group.

Another report from Nuffield Department of Population Health University of Oxford declared a large randomized controlled trial [Randomised Evaluation of COVid-19 thERapY (RECOVERY) Trial, 2020]. In this trial, there was no significant difference in the primary endpoint of 28-day mortality [25.7% HCQ vs. 23.5% usual care; hazard ratio 1.11 (95% confidence interval 0.98-1.26); p=0.10]. Meanwhile, there was also no evidence of beneficial effects on hospital stay duration or other outcomes. Subsequently, the authors discontinued randomization in the HCQ arm.

According to the latest news from WHO on 4^th^ July, the “SOLIDARITY” trial will discontinue the HCQ arm based on evidence from the current outcome from “SOLIDARITY” clinical trial (https://www.who.int/news-room/detail/04-07-2020-who-discontinues-hydroxychloroquine-and-lopinavir-ritonavir-treatment-arms-for-covid-19). The interim results do not provide solid evidence of increased mortality caused by HCQ, while some associated safety findings increased the deep concern about HCQ against COVID-19. WHO also announced that this decision applies only to the conduct of the Solidarity trial in hospitalized patients and does not affect the possible evaluation in other studies of HCQ in non-hospitalized patients or as pre- or post-exposure prophylaxis for COVID-19.

A randomized, double-blind, placebo-controlled trial emphasizing the effect of HCQ as postexposure prophylaxis from New England Journal of Medicine declared that the incidence of new illness compatible with COVID-19 did not differ significantly between participants receiving HCQ (11.8%, 49/414) and those receiving placebo (14.3%, 58/407) (Boulware et al., 2020). The treating regimen of HCQ was 800 mg once, followed by 600 mg in 6 to 8 h, then 600 mg daily for 4 additional days. The dosage in this trial was higher than those in China. The reasons might be conducted as two sides. The one reason might be different ethnicity in the patient population. In this trial, patients were across the United States and parts of Canada, which belongs to the white race. The other reason might be related with the average weight of recruited patients. Recommendations on screening for HCQ retinopathy by the American Academy of Ophthalmology were revised in 2016. These revised recommendations suggested that the maximum daily dose should be based on real body weight (Marmor et al., 2011).

To sum up, the higher dosage of HCQ might be brought out with more adverse reaction in clinic against COVID-19. The positive effect of HCQ on COVID-19 were controversial according to the current outcome.

### Potential Risk of HCQ in Clinic to Treat COVID-19

According to the usage of HCQ in clinic, the major risks of HCQ in clinic were retinal toxicity and ophthalmologic adverse symptom (Nika et al., 2014). Herein, renal impairment (such as eGFR <= 30 ml/min/1.73 m^2^) have been taken into consideration in the exclusion criteria among most of CQ-based and HCQ-based clinic trials, as exhibited in **Supplementary Tables 1** and **2**. Retinal toxicity from CQ and HCQ has been recognized for many years (Easterbrook, 1992). Although HCQ has been demonstrated less side effect compared to CQ *via* ocular administration, retinal toxicity to HCQ should not to be neglected owing to it might continue to progress even after cessation of therapy. What’s more, the increasing risk of visual loss were verified concerning with retinal toxicity (Michaelides et al., 2011).The retinal toxicity was associated with duration of HCQ use, daily HCQ dosage, and presence of kidney disease (Mititelu et al., 2013). Proper dosing of maximum 5 mg/kg of HCQ *via* oral administration was generally accepted (Kim et al., 2017). As aforementioned, the maximum daily dose should be based on real body weight (Marmor et al., 2011). The daily dosage of HCQ on lupus erythematosus (SLE) and rheumatoid arthritis (RA) were at much higher doses (up to 600 mg/d or 800 mg/d) to treat these autoimmune diseases (Al-Rawi et al., 2018). The clinical trials related with HCQ were a lot, while the therapeutic effect on virus was few. In1997, a randomized, double-masked trial in which HCQ (800 mg/d) or zidovudine (500 mg/d) was given to 72 HIV-l-infected patients for 16 weeks orally in New York (Sperber et al., 1997). The data have shown that no adverse reactions were observed in either the HCQ or zidovudine group in the study medications with levels of recoverable HIV-l RNA decreasing in both groups. Furthermore, the level of interleukin-6 and serum immunoglobulin G, which related with HIV-l replication in chronically infected cells and might be associated with the development of malignancies and autoimmunity, were significantly decreased in the HCQ treated group but not in the zidovudine group. Basing on the results of this clinic trial, the dosage of 800 mg/d for continuously 16 weeks medication of HCQ was well tolerated and HCQ suppressed HIV-l replication in infected patients.

A long-term, multinational, network cohort and self-controlled case series study has reported serious heart rhythm problems with HCQ, especially when taken at high doses or in combination with the antibiotic azithromycin (registration number: EUPAS34497). The study period started from 01/09/2000 and ended in 2020 with participants with a history of RA. The outcome could be drew as that short-term HCQ treatment is safe, while addition of azithromycin may induce heart failure and cardiovascular mortality, potentially due to synergistic effects on QT length. Herein, the authors call for caution if such combination is to be used in the management of COVID-19 (Lane et al., 2020).

Despite the adverse risk of HCQ, some reports have bared out HCQ showed protective effect on coronary artery, dyslipidemia, diabetes mellitus, preeclampsia, and chronic inflammatory diseases and so on. A clinical trial in Taiwan (registration number: CSMUH CS15134) confirmed that HCQ users versus non-users for coronary artery diseases events was 0.32 (95% CI, P <0.01) over up to 10 years (Hung et al., 2018). This study revealed that RA patients taking with HCQ could decrease coronary artery diseases risk, while the dosage of HCQ was not clearly. A double-blind, randomized, out-patient study (registration number: CTRI/2010/091/006138) in India which was conducted in 328 patients with primary dyslipidemia patients, was demonstrated that HCQ could emerge as a potential drug for combination with statins for treatment of dyslipidemia (Pareek et al., 2015). The benefits of HCQ combinational with rosiglitazone (Gerstein et al., 2006), metformin (Kitabchi et al., 2005), acarbose (Chiasson et al., 2002), indicated that a reduction in diabetes risk of up to 77% for patients, who had been taken HCQ for at least 4 years. HCQ was used at a low and fixed dose (200 mg/d) to ensure that the daily dose did not exceed 6.5 mg/kg. A latest retrospective cohort study conducted in 2019, aimed to investigate the impact of HCQ on pregnancy with SLE (Seo et al., 2019). This study included 151 pregnancies in 122 patients with SLE (80 pregnancies in the HCQ treatment group and 71 pregnancies without HCQ medication). The results revealed that preeclampsia was significantly less complicated (P=0.032) in the HCQ treatment group compared with HCQ non-treatment group. Moreover, the data showed pregnancy outcomes in SLE patients could be improved in the HCQ treatment group with about 90% reduction of preeclampsia. However, in this study, the specific dosage of HCQ was not supplied. Another large observational cohort study for patients with RA in China, the incidence rate of chronic inflammatory diseases was lower in HCQ users than in HCQ nonusers (P=0.01) with the prescribed daily dose (≦200, 201-400, or>400 mg) (Wu et al., 2018).

## Conclusions

Even though WHO declared the “SOLIDARITY” trial would discontinue the HCQ arm based on evidence from the current outcome from “SOLIDARITY” clinical trial to the conduct of the Solidarity trial in hospitalized patients and would not affect the possible evaluation in other studies of HCQ in non-hospitalized patients or as pre- or post-exposure prophylaxis for COVID-19, about 104 clinical trials are still ongoing in different countries to assess the potential effects of CQ and/or HCQ against COVID-19, according to the latest reports (Koschmieder et al., 2020). We may infer that although the clinic results are controversial of HCQ against COVID-19 currently, the scientists don’t give up the opportunities to explore the real mechanism, effect, and toxicity of HCQ. According to the research progress, the outcome of HCQ in clinic shows controversial results. We call for carrying out large-scale, medium- and long-term clinical trial of HCQ to verify whether HCQ being with the function against this undesired virus on human beings. Meanwhile, the lower dosage (200 mg/bid) of HCQ with short treating cycle (5 days) is worth to be considered for future studies.

## Author Contributions

YC conceived and wrote the paper. TS drew the figures. LZ contributed database tools. XD, TH, and ZL revised and edited the paper. HX conceptualization and supporting fund. All authors contributed to the article and approved the submitted version.

## Conflict of Interest

The authors declare that the research was conducted in the absence of any commercial or financial relationships that could be construed as a potential conflict of interest.
